# Pharmacological inhibition of NPY receptors illustrates dissociable features of experimental colitis in the mouse DSS model: Implications for preclinical evaluation of efficacy in an inflammatory bowel disease model

**DOI:** 10.1371/journal.pone.0220156

**Published:** 2019-08-01

**Authors:** Henry H. Ruiz, Stephanie Becker, Yu Bai, Luz A. Cortes-Burgos, Melissa M. Eckersdorff, Lynn E. Macdonald, Susan D. Croll

**Affiliations:** 1 Regeneron Pharmaceuticals, Neuroscience, Tarrytown, New York, United States of America; 2 The Graduate Center of the City University of New York, Graduate Program in Neuropsychology, New York, New York, United States of America; 3 Queens College of the City University of New York, Psychology, Flushing, New York, United States of America; Toho University Graduate School of Medicine, JAPAN

## Abstract

Administration of dextran sodium sulfate (DSS) to rodents at varying concentrations and exposure times is commonly used to model human inflammatory bowel disease (IBD). Currently, the criteria used to assess IBD-like pathology seldom include surrogate measures of visceral pain. Thus, we sought to standardize the model and then identify surrogate measures to assess effects on visceral pain. We used various 4% DSS protocols and evaluated effects on weight loss, colon pathology, biochemistry, RNA signature, and open field behavior. We then tested the therapeutic potential of NPY Y1 and/or Y2 receptor inhibition for the treatment of IBD pathology using this expanded panel of outcome measures. DSS caused weight loss and colon shrinkage, increased colon NPY and inflammatory cytokine expression, altered behaviors in the open field and induced a distinct gene metasignature that significantly overlapped with that of human IBD patients. Inhibition of Y1 and/or Y2 receptors failed to improve gross colon pathology. Y1 antagonism significantly attenuated colon inflammatory cytokine expression without altering pain-associated behaviors while Y2 antagonism significantly inhibited pain-associated behaviors in spite of a limited effect on inflammatory markers. A protocol using 7 days of 4% DSS most closely modeled human IBD pathology. In this model, rearing behavior potentially represents a tool for evaluating visceral pain/discomfort that may be pharmacologically dissociable from other features of pathology. The finding that two different NPY receptor antagonists exhibited different efficacy profiles highlights the benefit of including a variety of outcome measures in IBD efficacy studies to most fully evaluate the therapeutic potential of experimental treatments.

## Introduction

Inflammatory bowel disease (IBD) is characterized by chronic inflammation of the gastrointestinal (GI) tract and includes Crohn’s disease (CD) and ulcerative colitis (UC). Clinical manifestations include diarrhea, rectal bleeding/bloody stool, visceral pain and severe weight loss [1]. Histopathological analysis of tissue biopsies from humans reveals severe structural damage to the mucosa characterized by loss of goblet cells [2] (mucin-secreting cells in the colon that contribute to the intestinal mucosal barrier [3]), immune cell infiltration, and thickening of the GI muscle layers [4]. The exact cause of IBD remains unknown but notably, the incidence of IBD in Western countries has nearly tripled over past decades to an estimated 3 million affected adults [5]. Thus, IBD treatment represents an unmet medical need that warrants further pathophysiological investigation.

Several experimental models of IBD have been developed [6–9] including the dextran sodium sulfate (DSS)-induced model of colon pathology. The DSS model is one of the most widely used models for its simplicity, reproducibility, and ability to induce a pathological phenotype that mimics the clinical presentation of human IBD [10]. The magnitude of pathology varies greatly depending on the concentration and duration of exposure to DSS, which typically varies between concentrations of 3–5% DSS and exposures of 5–9 days in mice [11]. While the flexibility of the DSS model is one of its strengths, the lack of both a standardized DSS protocol and an in-depth characterization of its sequelae are gaps in the colitis literature. These gaps are particularly problematic when attempting to compare results from studies using different DSS protocol variations, especially in the context of assessing the therapeutic value of novel pharmacological agents.

DSS-induced weight loss, diarrhea, bloody stool, lethargy and colon shrinkage are reliably observed, and can be objectively quantified. However, these features vary as a function of both the DSS concentration used [12] and the exposure time. DSS-induced colitis most often presents as inflammation in the distal colon [13], similar to human UC [14], characterized by high expression of inflammatory cytokines (eg. tumor necrosis factor alpha [TNFα] [15, 16], interleukin-1 beta [ILβ] [17]), and neutrophil infiltration (often quantified by myeloperoxidase [MPO] as a surrogate measure). Histopathological analyses, when reported, rely on variations of previously proposed subjective measures [18–20], which are often quantified differently from one group to another. Fatigue and abdominal pain are two important human clinical symptoms that have been overlooked in most animal models of IBD, at least in part due to limited availability of simple methodologies for evaluating visceral discomfort in rodent models.

In the current studies, we sought to select and fully characterize a DSS protocol that closely models human IBD pathology in terms of gross pathology, histopathology, biochemistry (inflammatory and other markers), colon gene perturbations, and behavioral alterations. In addition to adapting and applying subjective measures for assessment of pathology, we used objective, quantitative measures whenever possible to complement and/or extend colon pathology evaluation. Furthermore, we conducted a detailed behavioral characterization of DSS-treated mice in the open field test, which could serve as a simple behavioral tool to assess sickness behavior and visceral-pain related behaviors that associate strongly with gene perturbations in the colon. Among our biochemical markers, we describe a marked upregulation of NPY in the colon of DSS-treated mice.

Neuropeptide Y (NPY) is a peptide widely expressed in the central (CNS) and peripheral nervous systems (PNS), and is synthesized and released by cells that, in the PNS, are predominantly sympathetic neurons. Sympathetic neurons are part of the autonomic nervous system (ANS), and ANS dysfunction may be an important contributing factor to the pathogenesis of IBD [21–23]. In fact, ANS dysfunction has been linked to higher health care cost and lower quality of life [24] in IBD, while pharmacological normalization of autonomic function has been linked to disease improvement [25]. Of note, the sympathetic and parasympathetic fibers that make up the ANS directly innervate the GI tract [26] and regulate intestinal function and gut immunity [27].

NPY signals through one of its five G-protein coupled receptors (GPCRs), Y 1,2,4,5,6 [28], to play a fundamental role in the regulation of immune responses (see Wheway et al. [29]), including in IBD. Whole body NPY knock out mice exhibited less inflammation and improved histological scores compared to control mice after exposure to a 3% DSS solution for 6 days [30]. NPY’s Y1 receptor has been implicated in DSS-induced pathology using genetically engineered mice, pharmacological blockade, and/or antisense-mediated inhibition in mice fed a 5% DSS solution for 7 days [31, 32]. However, all current studies assessing the potential role of Y1 receptor signaling in IBD have relied heavily on subjective measures that do not always correspond to objective measures [31, 32]. Furthermore, Y2 signaling is also implicated in immune response regulation [33], and to our knowledge its potential role in DSS-induced pathology has not yet been fully explored.

We therefore applied our detailed assessment tools to test the hypothesis that systemic suppression of NPY signaling via its Y1 and Y2 receptors would have measurable and potentially dissociable therapeutic potential for the treatment of IBD.

## Materials and methods

### Animals and experiments

All procedures were conducted in full compliance with state and federal animal welfare regulations and with the approval of Regeneron’s Institutional Animal Care and Use Committee. Seven experiments using a total of 280 (40 per experiment) adult C57Bl/6 male mice (Jackson Laboratories, Bar Harbor, ME) between 16–20 weeks of age and weighing at least 26 grams were used for these experiments. Animals were housed in groups of 5 per cage in a 12:12 hour light:dark cycle (lights on at 7:00) and were provided food and water *ad libitum*. All animals were acclimated to the housing colony and water bottles for at least one week before treatment initiation.

### DSS preparation and colitis induction

Dextran Sodium Sulfate (average molecular weight 9,000–20,000, Sigma-Aldrich) was mixed for at least two hours in water to yield a 4% DSS drinking solution. This solution was placed into 250mL glass bottles with metal spouts and was given to experimental animals *ad libitum* as a replacement for drinking water. Control mice received regular drinking water in similar glass containers. Fluid consumption was monitored and bottles were replenished as needed for the duration of the experiments. Depending on the DSS protocol being evaluated, mice were exposed to 4% DSS for 4 or 7 consecutive days with or without a 3-day reversal to regular drinking water prior to tissue collection.

### Body weight

All animals were weighed once daily for the duration of the experiments using a calibrated Ohaus CS200 scale (Ohaus Corporation, NJ). Body weight was recorded to the nearest tenth of a gram. Animal health was carefully monitored, and if weight loss was greater than 25% from starting body weight, or if animals appeared to be in very poor health (lethargic, failure to groom, piloerection, etc), they were humanely euthanized.

### Pharmacological treatment

#### NPY receptor antagonism

Systemic peripheral NPY Y1 receptor antagonism was achieved by once daily subcutaneous injection of the preferential Y1 inhibitor BIBP 3226 (Sigma Aldrich; also binds neuropeptide FF receptor (NPFF); 79 fold more specific than against NPFF 2 and >1000 against Y2, 4, or 5) reconstituted in distilled water (dH_2_0) to a final injectable dose of 1mg/kg or 3mg/kg. Control animals received a vehicle (dH_2_0) injection instead. Similarly, Y2 receptor antagonism was achieved by once daily subcutaneous injection of the selective Y2 inhibitor BIIE 0246 (Tocris, >650 fold selectivity over Y1, Y4, and Y5 receptors) reconstituted in a 12.5% DMSO solution to a final injectable dose of 10mg/kg. Control animals received a vehicle (12.5% DMSO in dH_2_0) injection instead. For Y1 and Y2 receptor combined antagonism, animals received two subcutaneous injections daily with each antagonist reconstituted as described above for the duration of the experiment. The two experimental groups receiving only one antagonist also received the corresponding vehicle control injections for the other antagonist.

### Open field

On the last day of each experiment, animals were placed in a *smart frame* open field system configured with the automated tracking software Motor Monitor (Kinder Scientific) for Windows (Microsoft). The 16” (length) x 16” (width) x 15” (height) Plexiglas open field chamber was placed into a frame containing two horizontal laser beam detection arrays. Eight animals counterbalanced across experimental conditions were tested concurrently for 60 minutes each in the fields. Responses were measured in five-minute intervals and a total count for each measure for the 60 minutes was calculated by summing the twelve five minute bins. The following measures were collected: basic movements (operationally defined as any horizontal beam cross), immobility time (operationally defined as a lack of horizontal and vertical beam crosses), fine movements (operationally defined as changes in body position not meeting criteria for ambulation, includes grooming and head movements), X+Y axis ambulation (operationally defined as a complete relocation of the animal’s body), rears (operationally defined as vertical beam crosses), rearing time (the time spent breaking vertical beams), rest time (operationally defined by a lack of beam crosses lasting longer than 15 seconds), total distance traveled (calculated from known distances between beams and total beam crosses), normalized rears (defined as the ratio of total rears to X+Y ambulation to serve as a control for immobility) and average rear duration (defined as the ratio of time spent rearing to total rears).

### Tissue collection

Animals were euthanized by carbon dioxide (CO_2_) asphyxiation. The large intestine was identified, dissected, cleaned in a neutral PBS solution, and dried on an absorbent tissue. Full colon lengths were measured in centimeters (cm) on a dissecting board with an engraved ruler (Fisher Scientific) and then tissues were weighed in a calibrated scale that displayed weights to the nearest hundredth of a gram. The large colon was then divided in half and labeled as proximal (closest to the cecum) or distal (closest to the anus). The distal colon section (~4cm in length) was further subdivided into three sections with the most distal 1–1.5cm of tissue used for histology, the adjacent 1–1.5cm of tissue used for immunoassays, and the remaining tissue used for gene profiling. Segments designated for histology were placed into tissue embedding cassettes (VWR) and left overnight to fix in 10% neutral buffered formalin. The inner segments were flash frozen in 200mL of 2-methylbutane (Fisher Scientific) on dry ice for immunoassay analyses and stored at -80° C. The segments designated for gene profiling were placed in 5ml RNA*later* tubes (Qiagen), stored overnight at 4° C, and then transferred to -20° C until processed.

### Tissue processing

#### Histology

Distal colon samples immersed in 10% neutral buffered formalin were washed at least three times with phosphate buffered saline (PBS) and immersed in 70% alcohol until processed for paraffin embedding. Samples were placed into an automated tissue processor (TBS, model ATP1) and allowed to process overnight. Tissues were then dehydrated through graded alcohols (70, 95, and 100%) and immersed in xylenes and wax twice each at 58° C. Samples were next placed in the liquid paraffin chamber of a tissue-embedding center (TBS, TEC-120). Once embedded, samples were mounted onto a frozen stage for at least one hour for paraffin solidification. Colon samples were then sectioned at 7μm thickness and collected in a 1:10 series onto slides. At least two slides, each with four or more sections, were collected per animal. Finally, each slide was stained using an Alcian Blue solution or a Carazzi’s hematoxylin and eosin (H&E) progressive method protocol for pathological assessment (Histoserv, Inc). Stained slides were imaged using an automated imager (Leica, Model SL801) at a maximum magnification of 40X.

#### Histopathological analysis

At least 4 images for each colon sample were captured and digitally stored. Using the publicly available imaging software Image J (NIH), five measurements of thickness or length, as relevant, were taken for each of the different anatomical areas of interest (colon muscle layers and colon crypts). With 4 images per sample, a total of 20 length measurements were collected for each measure per animal. H&E and Alcian blue-stained distal colon section images were scored for inflammation and damage by an experimenter blind to treatment condition. Inflammation was subjectively scored using a modified version of the scale published by Ten Hove et al. [20]. At least four different images from each animal at different levels of the distal colon were scored to obtain an average score per animal. Briefly, tissues were scored using the following criteria: a) percent of area involved on a scale of 0–4 where 0 = 0%, 1 = 0–10%, 2 = 10–20%, 3 = 20–50% and 4 = >50%, b) erosion and ulceration, c) crypt loss, d) number of follicle aggregates, e) monocyte infiltration, f) edema and g) goblet cell loss with *b-g* being scored on a 0–3 scale where 0 = none, 1 = weak, 2 = moderate and 3 = severe cell loss (See S2 Fig). Finally, to further validate the goblet cell subjective score findings, Image J software was used to count nucleated goblet cells and to measure goblet cell diameter.

### Immunoassays

Colon samples used for immunoassays were first pulverized and then homogenized in a buffer containing 150mM NaCl, 20mM Tris, pH 7.5, 1% Triton-X, and protease inhibitor tablets (1 tablet for every 25mL of buffer). For pulverization, samples were removed from the -80° C freezer and placed on dry ice. The Eppendorf tubes were then opened one at a time and the tissue was placed in the tissue compartment of an ice-cold stainless steel Bessman tissue pulverizer mortar (Spectrum Labs). Using a lead hammer, the tissue was completely pulverized (hammering approximately 8 times per sample). 1mL of homogenization buffer was added to the pulverized tissue. Using a dispensing ULTRA-TURRAX T-8 (IKA) instrument, the tissue was homogenized in three 10-second intervals separated by 10-second breaks during which the probe was placed in wet ice to prevent overheating. The homogenate was then centrifuged for 20 min at 4°C and 1400 revolutions per minute (RPM). Homogenized samples were separated in aliquots to prevent repeated freeze-thaw cycles and protein degradation. A 20μL aliquot was placed on wet ice for protein analysis using a Pierce bicinchoninic acid (BCA) protein assay kit (Thermo Scientific). The BCA protein assay kit was used following the manufacturer’s protocol. Briefly, 25μL of standards (ranging from 25–2000μg/mL) and colon samples were added to a non-binding 96-well round-bottom clear plate in duplicate. 200μL of working reagent (50 parts reagent A: 1 part reagent B) were added to each well and mixed thoroughly for 30 seconds on a plate shaker. The plate was then placed in an incubator at 37°C for 30 minutes. Following incubation, the plate was read in a SpectraMax M2e spectrophotometer and analyzed with SoftMax Pro 5.3 (Molecular Devices) using a wavelength of 562nm. Sample concentrations were then calculated in relation to the standard values.

Colon homogenates were used to quantify different proteins of interest using commercially available assays in accordance with manufacturer protocols. Neuropeptide Y protein (Phoenix Pharmaceuticals) and myeloperoxidase (Hycult Biotech) expression levels were quantified using an enzyme-linked immunosorbent assay (ELISA) and read in the spectrometer referenced above. Multiple inflammatory cytokines (GM-CSF, IFN-γ, IL-1β, IL-2, IL-4, IL-5, IL-6, IL-10, IL-12 [p40/p70] and TNF-α) were quantified in single samples using a 10-Plex Mouse magnetic capture bead cytokine panel Luminex (Invitrogen), read in a FlexMap 3D instrument and analyzed using xPONENT 4.2 (Luminex).

### Gene profiling

Distal colon samples submerged in RNA*later* were removed from -20° C and trimmed to a total weight of 33mg. After weighing, tissue samples were transferred into 1ml of TRIzol solution. A plastic tissue cutter was placed into each sample tube for tissue homogenization using an Omni AH96 homogenizing workstation (Omni International). Samples were homogenized at 20,000 rpm for 2 min. RNA was extracted using the MagMax Total RNA Isolation kit (Life Technologies) following the supplier’s provided protocol, and the resulting RNA was quantified using a standard protocol. Briefly, 2μl of RNase free water was added to each sample and then the sample was placed in a Nanodrop 8000 spectrophotometer (Thermo Scientific) for quantification. RNA quality was determined using a QIAxcel RNA quality control kit (Qiagen) following the manufacturer’s protocol.

#### RNAseq read mapping

Sequenced reads in Illumina Hiseq2000 image files (BCL files) were converted to FASTQ format via Illumina Casava 1.8.2. Reads were decoded and merged for each individual sample. The overall read quality per sample was evaluated with FastQC (http://www.bioinformatics.babraham.ac.uk/projects/fastqc/) to retain only samples with sufficient quality. Subsequently, a two-step hierarchical mapping strategy was employed to retrieve the raw read counts mapped to each gene. Reads in each sample were first mapped against mouse transcriptome (http://data/ncbi/GBK/mouse) using Bowtie (http://bowtie-bio.sourceforge.net/index.shtml) with two allowed mismatches. For each gene, reads mapped to the sense-strand exons of the gene were identified and counted. The resulting unmapped reads were then mapped against the genomic sequence of each gene (gDNA) by Bowtie using the same mapping parameters. The reads mapped to the sense-strand introns were also added to the counts for each gene.

### Human IBD genetic data sets

We retrieved 18 curated biosets from 11 published studies from NextBio (https://www.ncbi.nlm.nih.gov/pubmed/20927376), each of which contains a list of publicly available gene signatures in colon that differentially express between the Crohn’s disease and/or ulcerative colitis patients and healthy controls or healthy tissue. Two of the 11 studies were conducted in pediatric patients and the other 9 in adults. One was conducted in adult twins discordant for IBD. All included both male and female subjects, and group sizes ranged from 5 to 60 in the 11 studies. We filtered each of the 18 lists to genes with a fold change of at least 1.5 in either the up or down regulated direction, as well as a p value of no more than 0.05. The filtering criteria is recommended by the MicroArray/Sequencing Quality Control society for gene signature derivation [34]. The 18 filtered gene lists were used to derive the metasignatures of IBD. A gene was considered to be part of the metasignature if it was perturbed in at least 9 of the 18 lists, with a mean fold change > = 2, and consistently in the up or down direction.

### Statistical analysis

All quantitative analyses and graphical representations were computed using either the Statistical Package for the Social Sciences (SPSS, Version 20 for Mac) or Graphpad Prism (Prism 8.2 for Windows) unless otherwise specified. Data points that were 2 or more standard deviations away from the mean in either direction and/or were identified as outliers by the Grubbs’ test were excluded from statistical analyses. All tests were conducted as two-tailed analyses with p<0.05, determined *a priori*, as the threshold for statistical significance.

For comparisons between two independent groups (eg. water vs. DSS), statistical differences between the sample means were evaluated using Student’s unpaired t-test. When the assumption of equal variances between the data sets was not met as determined by the F-test for equality of two variances, data were instead analyzed using the Mann-Whitney test for nonparametric data.

For comparisons among more than two group means with one or more independent variables, analyses of variance (ANOVAs) were performed. Specifically, body weight data were analyzed using mixed Factorial ANOVAs with time as a repeated measure and were tested for sphericity using the Geisser Greenhouse Epsilon value. Other comparisons among more than two groups across two independent variables were evaluated using two-way independent groups Factorial ANOVAs (eg. DSS treatment x drug treatment) with equality of variances assessed using the Brown-Forsythe Test. For one experiment evaluating more than two groups across one independent variable (drug treatment, S5 Fig), a one-way independent groups ANOVA was used. When variances were statistically different, data were analyzed using the Kruskal-Wallis test for nonparametric data. Pairwise differences between groups in ANOVAs were determined by *post hoc* analyses using Tukey tests. A detailed table of the statistical results is presented in S7 Fig. All graphs are shown as group means ± the standard error of the mean (SEM) unless otherwise stated with the number of samples/subjects (“n”) displayed on the bar graphs. Asterisks indicate differences between control and treatment groups while pound signs indicate differences between vehicle and drug treatment within the DSS-treated condition.

### Statistical analysis of differentially expressed RNA

Read counts summarized at the gene level represented the raw gene expression measures. An empirical minimum read count of 10 was applied to flag the “absence” and “presence” of genes in each sample, assuming the quantitation of a gene with fewer than 10 mapped reads would not be reliable. We normalized the raw gene expression in each sample by global scaling to match the median library size (i.e. the total number of mapped reads) as well as the 75% quartile of the gene-level read counts across all samples, as described in previous studies [BMC Bioinformatics 2010, 11:94]. For each comparison between two groups of samples, we first eliminated genes that were not flagged as “present” in all samples of the higher expressing group, resulting in on average about 13,000 out of total 35,161 genes for subsequent analysis. Next, fold changes associated with the comparison were calculated as the ratio between the arithmetic mean expression in the two groups. The statistical significance (p-value) of the differential expressions was assessed under the negative binomial distribution models using DESeq package (version 1.6, Genome Biology 2010, 11:R106). We selected genes with fold changes no less than 1.5 in either direction with p-values of at least 0.01 as the significantly perturbed genes. The final number of perturbed genes in the present work ranges from a few hundred to a couple thousand.

## Results

### Duration of 4% DSS exposure affected body weight loss patterns

**Fig 1:**
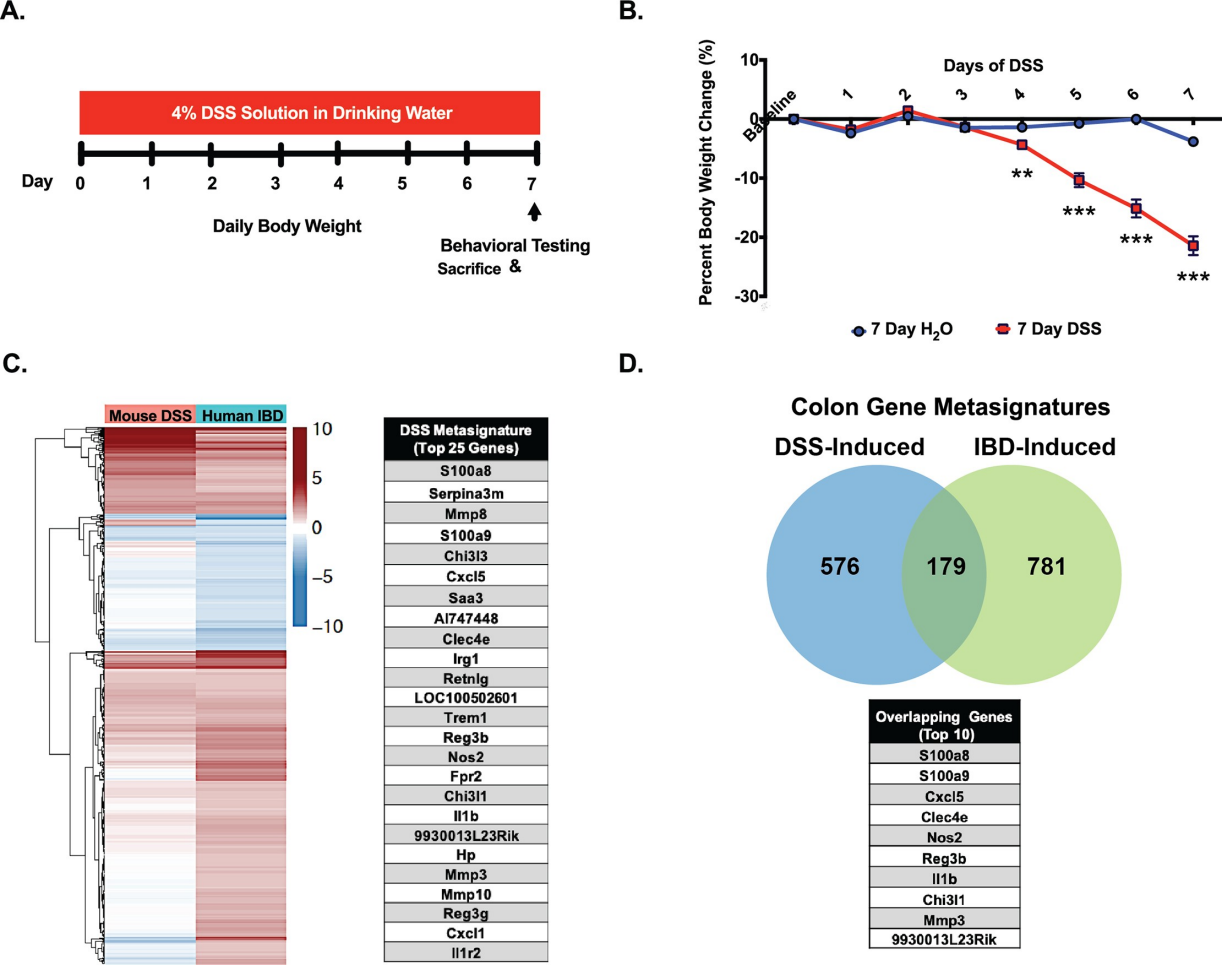
The 7-day DSS treatment protocol caused significant weight loss and a distinct colon gene metasignature that closely paralleled that observed in human IBD. A) Schematic representation of the experimental timeline. B) Percent body weight change from baseline after 4% DSS initiation C) Heat map of genes altered in the colon of mice after 7 days of DSS compared to gene alterations in human IBD, highlighting the top 25 genes in the metasignature. D) Venn diagram depicting the overlap in genes altered by DSS and human IBD along with a list of the top 10 overlapping genes. Asterisks correspond to p-values as follow: **p<0.01 & ***p<0.001.

We conducted a series of experiments in parallel comparing commonly used protocols with and without reversal to regular drinking water (see Fig 1A and S1A Fig for experiment schematic). Administration of a 4% DSS solution to male C57Bl/6 mice consistently induced significant body weight loss by the fourth day. Reverting mice back to tap water for 3 days after a 4 day exposure to DSS did not prevent further weight loss whereas reversal to water after 7-days of DSS exposure consistently halted the progressive weight loss (Fig 1B and S1B Fig). Thus, weight loss varied as a function of the DSS protocol variation used.

#### 7-day administration of a 4% DSS solution induced colon gene changes that paralleled those observed in colons from humans suffering from IBD

DSS induced a distinct pattern of gene regulation in colon tissue composed of 927 (of which 755 have human orthologs) significantly regulated genes. Comparison of the significant DSS gene changes with those obtained from human subjects suffering from IBD (which exhibited 1023 gene changes, of which 960 have mouse orthologs) revealed a significant overlap in altered genes (Fig 1C). Specifically, 179 genes were similarly affected by DSS and IBD (Fig 1D). Further analysis revealed that 7 days of DSS exposure yielded a more similar gene expression pattern to human IBD than reversion to water for 3 days. Thus, data from the 7-day DSS only group were combined with public human mixed IBD data to create an inflammatory colitis gene metasignature. Of note, immune-related genes predominated the colitis metasignature.

#### 7-Day DSS administration caused gross and micro-pathology of the colon similar to what is observed in human patients suffering from IBD

**Fig 2:**
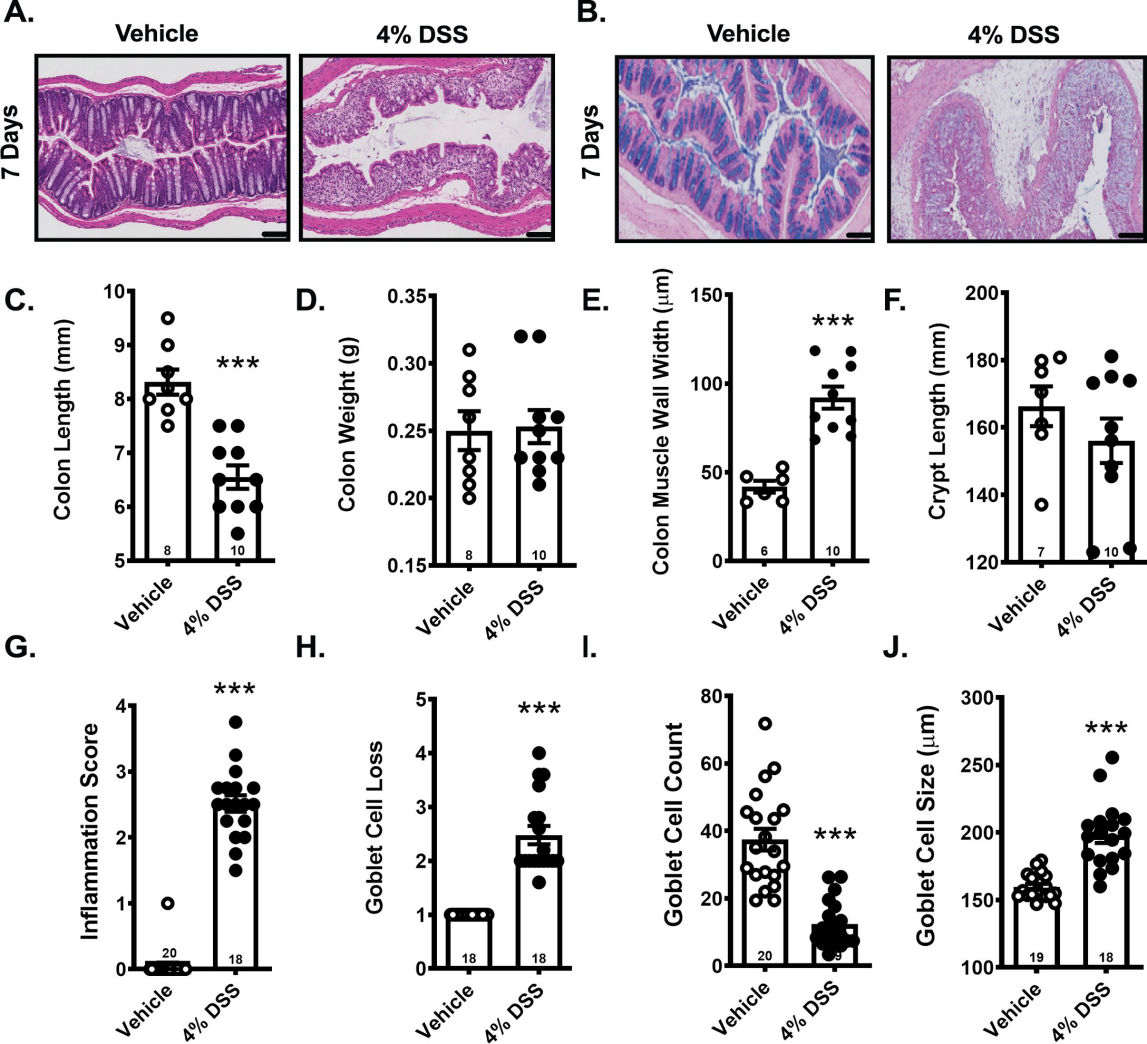
Exposure to DSS for 7 days caused colon shrinkage, muscle wall thickening, goblet cell loss and significant colon inflammation. A) H&E and B) Alcian blue-stained distal colon sections. C) Colon length, D) weight, E) muscle wall width and F) mucosa/crypt layer size. G) Subjective inflammation by H&E and H) subjective goblet cell loss scores from Alcian blue-stained sections. I) Goblet cell count per region of interest revealed a significant loss and J) increased average cell size after DSS exposure. Data are reported as group means ± SEM, ***p<0.001. Sample size for each group is indicated within its bar on the bar graph.

Distal colon samples from mice exposed to DSS for 7 days were stained with H&E (Fig 2A), and with Alcian blue to assess morphology and goblet cell presence (Fig 2B). 7-day DSS exposure resulted in significant colon length shrinkage (Fig 2C) while colon weight remained unaffected (Fig 2D), likely due to intestinal edema. Analysis from H&E-stained slides revealed significant thickening of the colon muscle wall (Fig 2E), similar to observations in human IBD. While DSS caused obvious damage to the colon mucosa structure (Fig 2A and 2B), the overall thickness of the mucosal layer was unaltered by DSS exposure (Fig 2F). We used an inflammation and goblet cell damage rating scale (scoring methodology detailed in S2 Fig) that, when scored by an experimenter blind to the experimental condition, consistently identified both colon inflammation (Fig 2G) and goblet cell loss (Fig 2H). Confirming the subjective rating, goblet cell counts were significantly lower after DSS (Fig 2I) while goblet cell size was increased (Fig 2J) in those cells that remained.

Gross colon anatomy and colon pathology was altered in the other DSS protocols evaluated and did not differ substantially from that achieved with the 7 day exposure protocol (refer to S1 Fig).

#### DSS-induced decreases in rearing behavior correlated strongly with the inflammatory colitis gene metasignature and may serve as a surrogate measure for visceral pain

**Fig 3:**
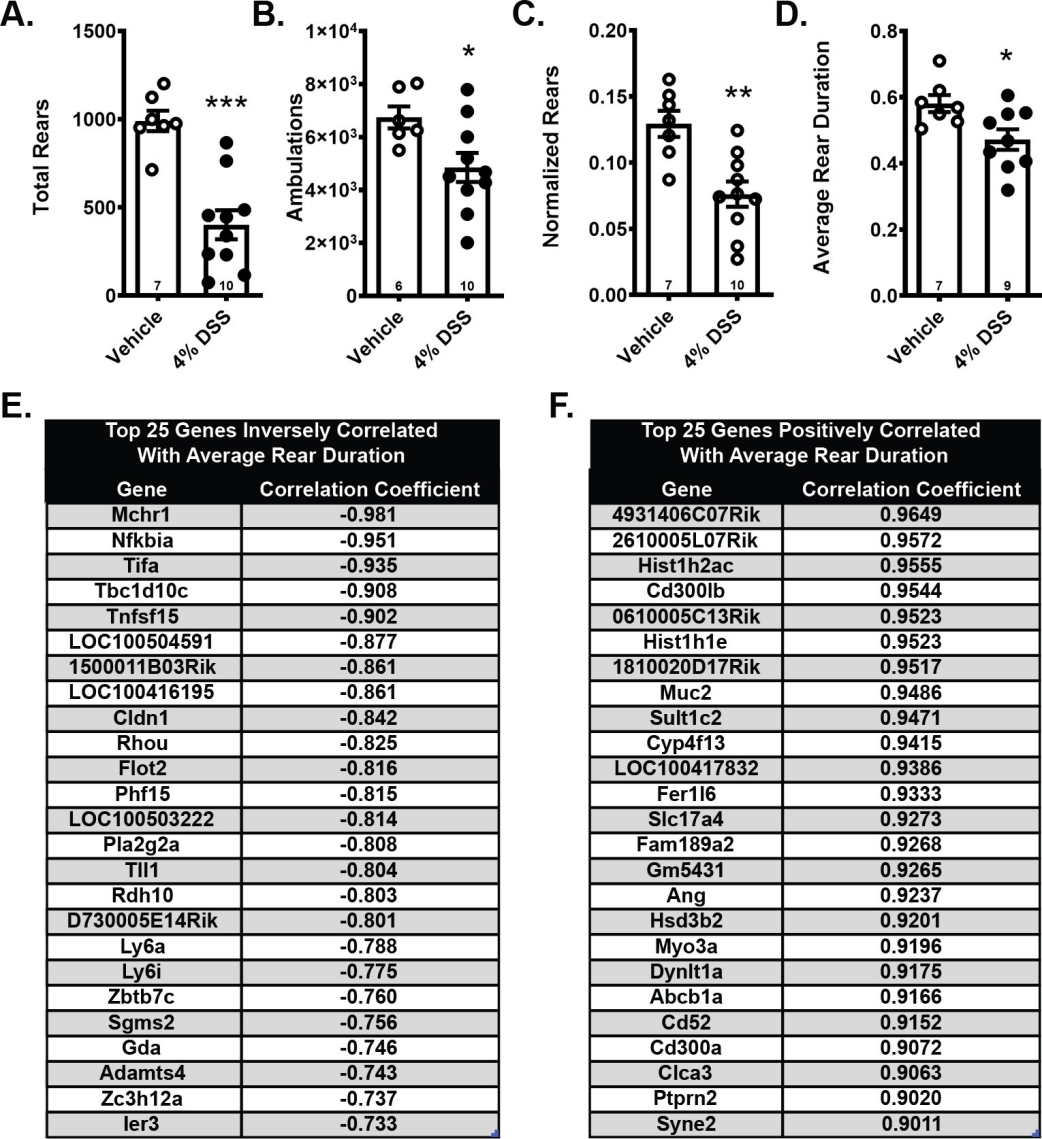
DSS exposure for 7 days suppressed mobility and rearing behavior in the open field test with average rearing time strongly correlating with altered colon genes. A) Total number of rears and B) ambulations were significantly decreased after DSS exposure. C) Normalizing rears to total movement did not diminish this decrease. D) Average time spent in each rear (rear duration) was also decreased by DSS. Top 25 DSS altered genes E) negatively and F) positively correlated with the average rear duration. A-D) Data are reported as group means ± SEM, *p<0.05, **p<0.01, ***p<0.001. Sample size for each group is indicated within its bar on the bar graph.

To test the hypothesis that DSS administration causes detectable visceral pain, we evaluated mouse behavior in the open field test as a potential surrogate measure of visceral discomfort. Rearing is an exploratory behavior commonly elicited in the open field, in which mice stand on their hind legs, stretching their abdominal area. Mice exposed to 7 days of DSS exhibited a significantly reduced number of total rears (Fig 3A) as well as a decrease in ambulatory locomotion in the field (Fig 3B). Because it is possible that the decrease in these behaviors represented a general decrease in mobility and exploratory behavior due to sickness behavior, we also normalized total rears to the total ambulation for each mouse. Using normalized rears, we still observed a significant decrease in rearing in the DSS exposed mice (Fig 3C). This finding suggests that the decrease in rearing behavior cannot be fully explained by a decrease in general activity, and we propose that the abdominal stretch induced by rearing is aversive to animals experiencing visceral discomfort. Average rearing duration was also evaluated, because shorter rears may indicate a reluctance to engage in prolonged abdominal stretch. Indeed, rearing duration was significantly shorter in mice exposed to DSS (Fig 3D), and may provide a simple surrogate measure of abdominal discomfort. Rearing duration correlated strongly with colon genes altered by DSS. We present the top 25 genes that were negatively (Fig 3E) and positively (Fig 3F) correlated with average rear duration. Of note, none of these DSS-Induced behavioral alterations were observed after 4 days of DSS whereas mice exposed to the 7-day DSS protocol and then reverted to water continued to exhibit decreased rearing behavior (data from other protocols included in S3 Fig). Together, our data suggest that open field behavior varies as a function of DSS protocol used, with rearing behavior emerging as a potential behavioral surrogate measure for visceral pain in the 7-day DSS models.

#### DSS exposure induced a reliable inflammatory signature in the distal colon

**Fig 4:**
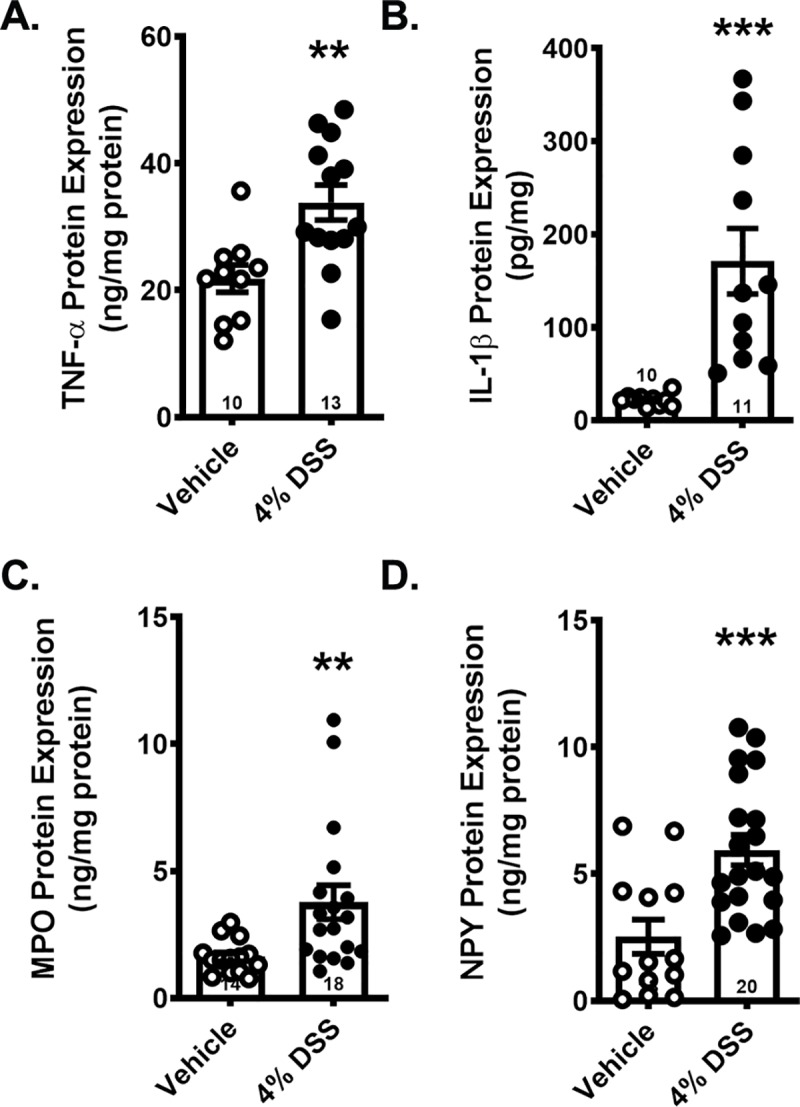
Exposure to DSS for 7 days caused colon inflammation characterized by increased expression of proinflammatory cytokines and concomitant NPY overexpression. Expression levels of A) TNFα, B) IL-1β, C) MPO and D) NPY in colon tissue. Data are reported as group means ± SEM, **p<0.01, ***p<0.001. Sample size for each group is indicated within its bar on the bar graph.

DSS exposure led to significantly higher expression of TNF-α (Fig 4A) and IL-1β (Fig 4B) in colon tissue. Significant overexpression of TNF-α occurred as early as day 4 (S4A Fig) while increases in IL-1β were more pronounced after 7 days of DSS administration (S4B Fig). Colon MPO levels, which are commonly used to assess neutrophil infiltration in the DSS model, tended to be higher in DSS-exposed mice by day 4 (S4C Fig), but only reached statistical significance by day 7 (Fig 4C). Colon NPY expression was significantly higher in mice exposed to DSS for 7 days (Fig 4D), but was not significantly elevated with shorter DSS exposure times (S4D Fig).

#### Systemic inhibition of the Y1 receptor with BIBP 3226, but not the Y2 receptor with BIIE 0246, significantly altered the DSS gene metasignature in the colon despite attenuated weight loss with both treatments

**Fig 5:**
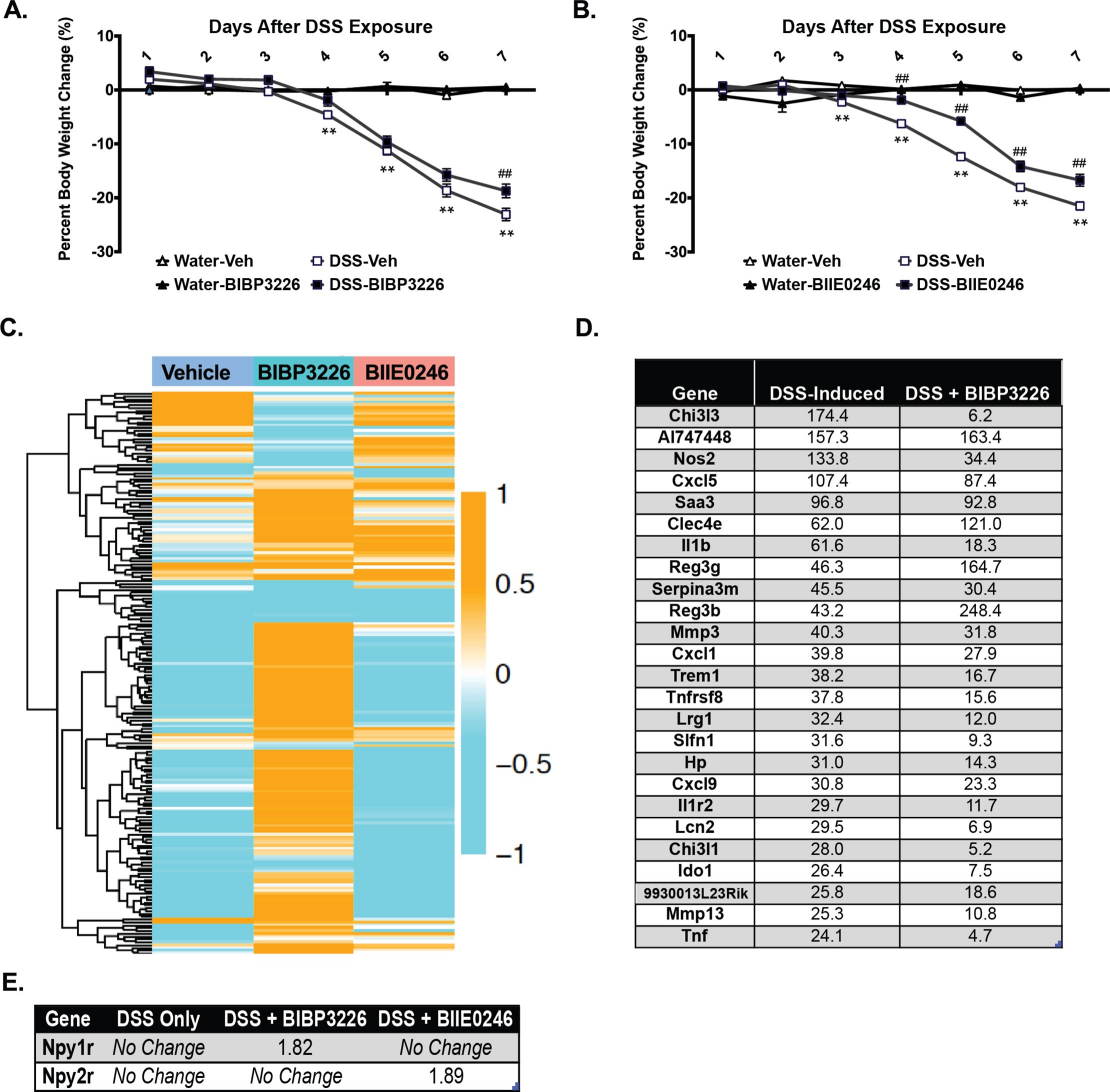
Systemic inhibition of NPY signaling via its Y1 or Y2 receptors slightly, but significantly, slowed down DSS-induced weight loss progression and Y1, but not Y2, receptor inhibition altered DSS-induced colon gene changes. Percent body weight change for control and DSS exposed mice treated with A) the Y1 (BIBP 3226) or B) the Y2 (BIIE 0246) receptor inhibitor. C) Heat map of gene alterations in the DSS metasignature induced by the systemic inhibition of Y1 (BIBP 3226) and Y2 (BIIE 0246) receptors during 4% DSS exposure for 7 days suggest effect on gene expression by BIBP 3226 but not BIIE 0246. D) Top 25 genes from the DSS-induced metasignature that were significantly regulated by systemic Y1 (BIBP 3226) receptor inhibition. E) NPY receptor gene expression in colon tissue after inhibitor treatment showed potential compensatory changes in expression. A-B) Data are reported as group means ± SEM, **/## p<0.01.

Systemic inhibition of the NPY Y1 receptor with BIBP 3226 (Fig 5A) and to a larger extent Y2 inhibition with BIIE 0246 (Fig 5B), significantly slowed the progression of DSS-induced weight loss, suggesting a potential role for NPY receptor signaling in this aspect of DSS-induced disease. Next generation gene sequencing revealed that systemic inhibition of Y1 receptors with BIBP 3226, but not Y2 inhibition with BIIE 0246, resulted in a colon gene metasignature that differed significantly from that induced by DSS alone (Fig 5C and 5D). Because the gene metasignature appeared to be driven largely by inflammatory genes, these data suggest that Y1 signaling, but not Y2, regulates DSS-induced inflammation. Of note, transcript levels for the Y1 (Npy1r) and Y2 (Npy2r) receptors in the colon were upregulated in mice treated with BIBP 3226 and BIIE 0246, respectively (Fig 5E), likely as a compensatory response to systemic receptor inhibition.

#### Systemic inhibition of neither Y1 nor Y2 prevented DSS-Induced gross or micro-pathology of the colon

**Fig 6:**
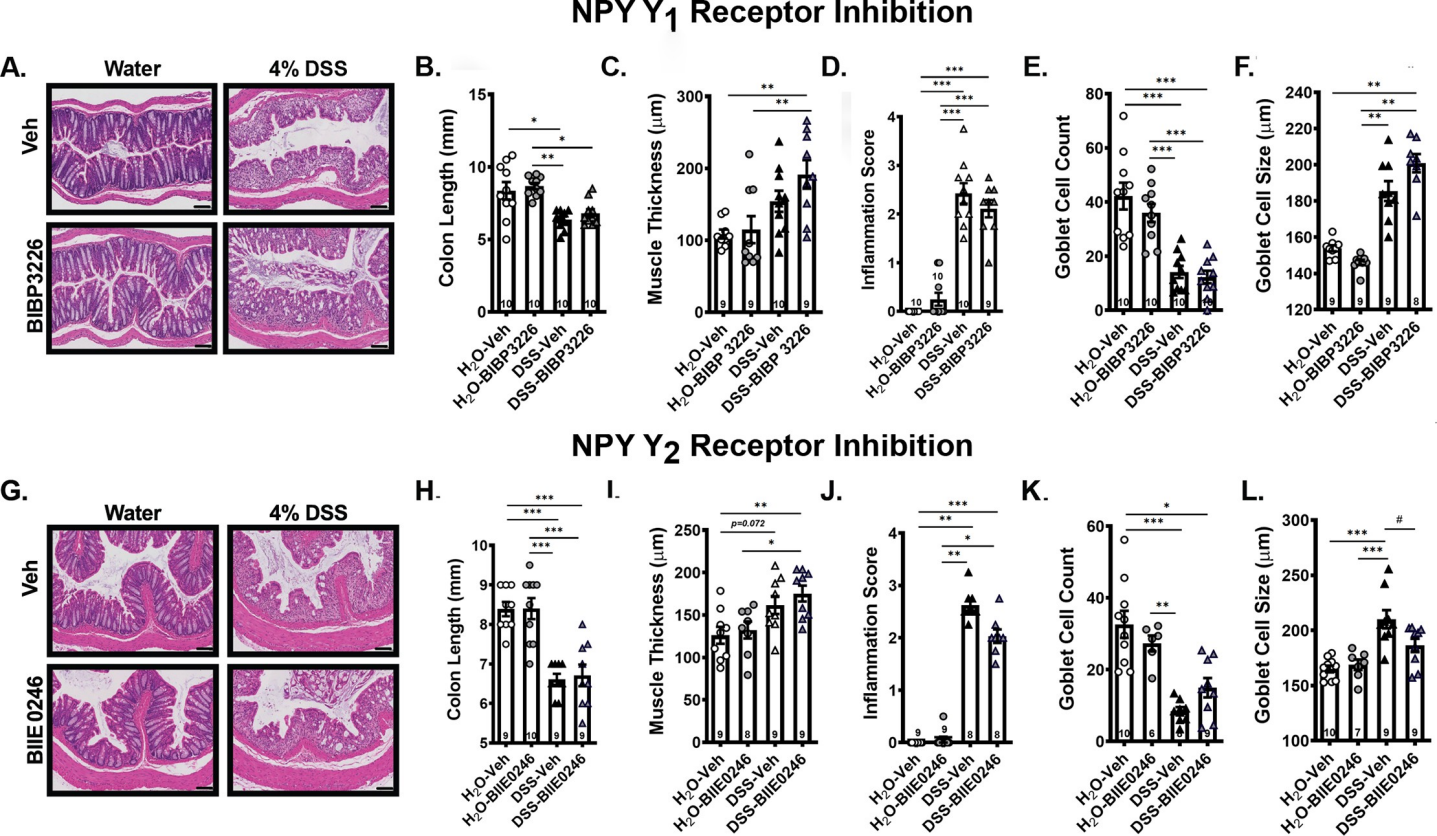
Inhibition of neither the Y1 nor Y2 receptor prevented DSS-induced colon histopathology. A) & G) Distal colon sections stained with H&E for mice treated with the Y1 versus Y2 receptor inhibitor showed no effect of receptor antagonists. B) Colon length from vehicle versus DSS exposed mice treated with the Y1 versus H) Y2 receptor inhibitor showed no effect of inhibitors. No effect of C) Y1 or I) Y2 receptor inhibition on distal colon muscle wall thickness. D) & J) Subjective inflammation scores from H&E-stained distal colon sections were unchanged by inhibitors. Goblet E) cell count and F) size in DSS exposed mice treated with the Y1 versus Y2 receptor inhibitor (K & L) revealed small and opposite changes in goblet cell size but not count with receptor inhibitors. Data are reported as group means ± SEM. Asterisks correspond to p-values as follow: */# p<0.05, **p<0.01, ***p<0.001. Sample size for each group is indicated within its bar on the bar graph.

Neither Y1 nor Y2 receptor inhibition prevented mucosal damage (Fig 6A and 6G), colon shrinkage (Fig 6B and 6H), muscle wall thickness changes (Fig 6C and 6I), colon inflammation score (Fig 6D and 6J) or goblet cell count (Fig 6E and 6K) induced by DSS. In contrast, inhibition of Y1 receptors did not prevent DSS-induced goblet cell enlargement (Fig 6F) whereas Y2 receptor inhibition resulted in significantly smaller goblet cells (Fig 6L).

#### Y2 receptor inhibition increased rear duration in mice exposed to DSS

**Fig 7:**
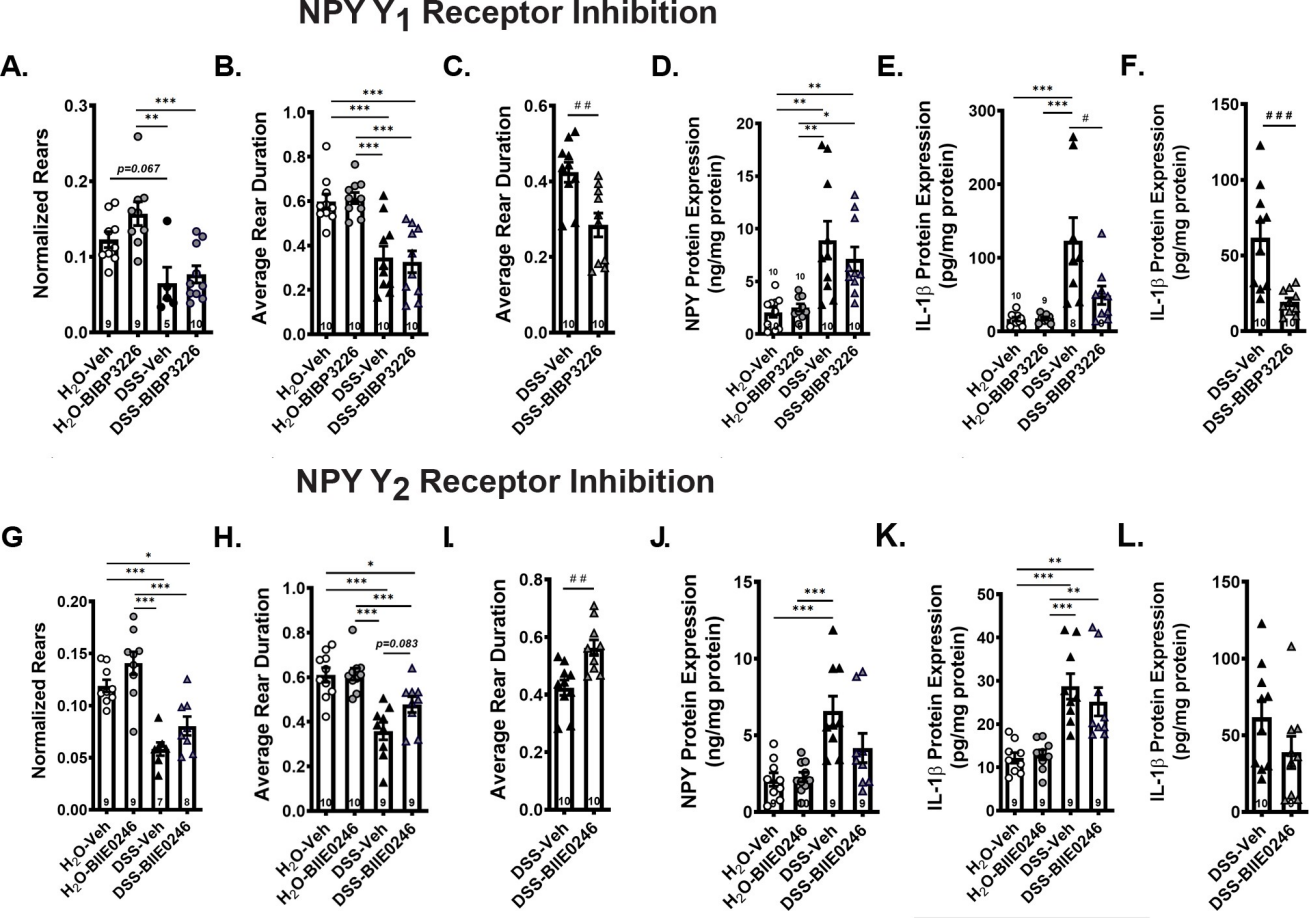
NPY Y2 receptor inhibitor improved rearing duration while Y1 receptor inhibition suppressed inflammation in DSS-exposed mice. Rears normalized to total ambulation (A & G) were unaffected by either inhibitor treatment, but average duration per rear (B shows Y1; H shows Y2) was improved by Y2 receptor inhibition. Data from a repeat experiment showed that the improvement in rear duration with Y2 inhibitor treatment (I) replicated, but improvement was still not observed with Y1 treatment (C). Distal colon expression levels of NPY for control and DSS exposed mice treated with the D) Y1 (BIBP 3226) and J) Y2 (BIIE 0246) receptor inhibitors showed no significant impact of NPY receptor inhibitors on NPY release in colon. Colon IL-1β expression was significantly decreased by the Y1 (E) but not the Y2 (K) inhibitor. A repeat experiment verified the consistency of the significant decrease in IL-1β with Y1 (F) but not Y2 (L) receptor inhibition. Data are reported as group means ± SEM. Significant p-values are illustrated as follow: */# p<0.05, **p<0.01, ***/### p<0.001. Sample size for each group is indicated within its bar on the bar graph.

Preventing NPY signaling via the Y1 or Y2 receptors did not affect the total number of rears or total ambulations measured after DSS or water (data included in S6 Fig). In addition, neither treatment altered the ratio between the number of rears by total ambulations after DSS (Fig 7A and 7G). However, Y2 receptor inhibition resulted in a statistical trend toward longer rears (Fig 7H). To determine if this effect was consistent, a second study was conducted (full results in S5 Fig), and this study revealed a significant alleviation of the rearing impairment with Y2 inhibition, suggesting replicability of this finding in spite of its small effect size (Fig 7I; Fig 7C shows rpeat for Y1 inhibitor for comparison). The second study contained a pilot arm in which inhibitors were combined, and co-treatment with both inhibitors did not reverse DSS-induced decreases in rearing behaviors (data included in S5 Fig). These data suggest that suppressing NPY signaling via the Y2 receptor does not affect DSS-induced colon morphology or inflammation but may, to some degree, attenuate the associated visceral discomfort.

#### Inhibition of Y1, but not Y2, suppressed DSS-induced colon IL1β overexpression

In line with our previous findings, mice exposed to DSS exhibited significantly higher levels of inflammatory proteins including TNF-α, IL-1β, MPO as well as increases in NPY levels in the distal colon. Neither Y1 nor Y2 inhibition significantly altered NPY levels (Fig 7D and 7J). Inhibition of Y1 receptors did not significantly alter MPO or TNF-α levels (data included in S6 Fig), but significantly suppressed the expression of IL-1β (Fig 7E; Fig 7F shows replication from a second study). In contrast, the inflammatory profile in mice treated with the Y2 receptor inhibitor was comparable to that of DSS exposed mice treated with a vehicle solution (IL-1β Fig 7K; Fig 7L is from a second experiment; full additional data set shown in S6 Fig). Analysis of tissues from a pilot combined treatment experiment showed that combined treatment with both inhibitors did not potentiate the anti-inflammatory effects of Y1 inhibition alone (data included in S5 Fig).

## Discussion

The DSS model is among the most commonly used experimental models of inflammatory bowel disease (IBD) due to its simplicity and its ability to induce pathology similar to what is observed in human patients. However, multiple variations of this model have been published using various concentrations and durations of DSS, making it difficult to compare results across studies. In addition, few studies have been published that include the full range of relevant outcomes, with a notable deficiency in the evaluation of visceral pain or sickness-related behaviors, a major contributor to reduced quality of life in human patients.

We compared commonly used variations of the DSS model to determine which protocol best modeled aspects of human disease. We observed unexpected differences between protocols in outcomes such as body weight loss, colon histopathology, and colon gene alterations that may explain some of the discrepancies in the literature. For example, we found that compared to 7 days, 4 days of DSS exposure leads to less weight loss and no colon shrinkage, but more overall colon damage and inflammation. When animals are reverted back to water, those exposed to 7 days of DSS stopped losing weight while mice exposed to 4 days of DSS continued to progressively lose weight. We report that both DSS protocols tested yielded a consistent DSS-induced metasignature of genes altered in the distal colon, consistent with previous studies. However, exposure to a 4% DSS solution for 7 days gave rise to a more robust metasignature characterized by 927 perturbed genes (755 with human orthologs) of which 179 overlapped with those observed in human IBD. Thus, we selected the 7 day protocol for the remainder of our studies.

Our finding that C57Bl/6 mice with experimental colitis exhibit decreased rearing, rearing duration, and ambulation in the open field suggest the possibility that behavioral assays could be used to evaluate discomfort with various treatment paradigms. While reduced overall activity is observed in many diseases and probably reflects sickness behavior, we were able to determine that rear duration and rears normalized to overall ambulation could potentially represent surrogate measures of visceral pain. We reasoned that gastrointestinal discomfort could inhibit animals from stretching their abdomens, especially for long periods of time. We are hopeful that open field assessments will provide a tool for evaluating visceral nociception and for differentiating between treatments for IBD discomfort (visceral pain and sickness behavior) versus pathology. In humans, visceral pain is not necessarily relieved with resolution of colon inflammation [35] and chronic narcotic treatment is often needed by IBD patients [36] to control their pain. Our data suggest the possibility that different treatments could be evaluated and then tailored to each IBD complaint, thus providing the most complete therapeutic regimen for patients.

Our data suggest that rearing behaviors may be able to selectively unveil therapeutic strategies for relieving abdominal discomfort dissociable from those relieving pathology. Specifically, we observed that inhibition of NPY Y2, but not Y1, receptor signaling increased the average time spent rearing, suggesting a beneficial effect on visceral discomfort. Interestingly, weight loss, which is a common measure of response to treatment in experimental colitis, is more attenuated by Y2 than Y1 inhibition, in concordance with effects on rear duration. In contrast, inflammation, the most common target of human therapeutics for IBD, is instead more attenuated by Y1 receptor inhibition, albeit without colon structural protection. This potential mechanistic dissociation between quality of life factors such as weight loss and behavior, and inflammation suggests that targeting inflammation alone may not treat all aspects of the patient experience of IBD.

The mechanistic basis of the differential profile of effects of inhibiting NPY signaling through these two receptors is not well-understood. We chose to evaluate the role of NPY signaling in the DSS model based both on our finding of elevated colonic NPY in the DSS model and on information published in the literature. Prior publications have reported that sympathetic responses are altered in IBD, that sympathetic nerves innervate and regulate colon function [37], and that NPY, a key sympathetic neuroregulator, could play a role in the etiology of DSS-induced colitis [38].

While previous studies have reported amelioration, attenuation, and modulation of DSS-induced colon structural damage and inflammation by pharmacologically or genetically inhibiting the NPY system systemically [30, 31, 39], our findings suggest that Y2 receptor inhibition does not alter any of the traditionally evaluated aspects of DSS-induced colitis and Y1 inhibition failed to protect against colon structural damage. This discrepancy in observation could potentially be explained by the different DSS protocols used in those studies and/or the different pathology scales, but could also be due to other differences in methodology, including the specific pharmacological intervention used. Our data do support the notion that Y1 inhibition results in decreased DSS-induced inflammation, but our experimental design did not allow us to establish the mechanism for this effect. Because NPY Y1 receptors play a key role in the regulation of immune cell activity including activation, mobilization and adhesion [29, 33, 40], it is possible that systemic inhibition of this receptor during DSS administration suppressed colon cytokine levels via direct action on immune cells. Further research will be necessary to elucidate the underlying mechanism of the effect. Y2 receptor signaling has also been implicated in the regulation of immune responses [41], and we therefore inhibited this receptor and assessed its effect on DSS-induced pathology. However, we failed to demonstrate any significant anti-inflammatory effects of Y2 inhibition, and instead provide evidence that Y2 receptor signaling may play a role in promoting IBD-related visceral pain.

In summary, our work evaluated multiple features of DSS-induced colitis in C57Bl/6 mice, including behavioral measures that could potentially be used to evaluate sickness behavior and visceral discomfort. The addition of these measures to the assessment of colitis severity in experimental models may provide a powerful tool for assessing the full therapeutic potential of an agent for the treatment of all aspects of IBD. Future work could evaluate this expanded panel in other disease models or strains of mice, as well as in female mice, to evaluate whether these effects are specific to the DSS model and/or to male C57Bl/6 mice. In our initial evaluation of a therapeutic approach using this expanded panel of outcomes, we examined the therapeutic potential of NPY receptor Y1 (BIBP 3226) and Y2 (BIIE 0246) inhibitors. We observed a dissociation of drug effects, such that BIBP 3226 suppressed increases in IL-1ß while BIIE 0246 improved rearing duration and caused attenuated weight loss. This potential dissociation between quality of life factors such as behavioral function and general health and inflammation suggested that different therapeutic strategies might be warranted for each of the key features of colitis.

## Conclusions

Administration of DSS to male C57Bl/6 mice produces acute, chronic and relapsing models of human IBD. While this is a widely used experimental model, standardized protocols that allow for comparison among independent studies are lacking, as are surrogate measures of visceral pain, a hallmark of human IBD that results in reduced quality of life for patients. Current treatments target the inflammatory response but do not prevent colon damage nor alleviate visceral pain. Here, we characterized and compared, in parallel, the pathology induced by four commonly used DSS protocol variations consisting of 4% DSS administration for 4 or 7 days with and without a 3 day recovery period. We found differences in disease profile depending on the DSS model used. RNA sequencing studies revealed that a 7 day DSS protocol induces a colon gene metasignature with significant overlap to the gene changes observed in colon samples from humans suffering from these diseases. Notably, we identified mouse rearing behaviors in the open field test as potential surrogate measures for visceral pain related to IBD. Inhibition of NPY signaling has been proposed as a potential therapeutic strategy for IBD but results are inconsistent. We tested antagonists of two different NPY receptors, Y1 antagonist BIBP 3226 and Y2 antagonist BIIE 0246, using an extensive panel of outcome measures. Pharmacological inhibition of these two NPY receptors showed different profiles of effect, with Y1 inhibition reducing inflammation and Y2 inhibition slowing the progression of DSS-induced weight loss and improving rearing behavior. Neither treatment improved colon structural damage. Taken together, these results suggest that evaluating an expanded profile of outcome measures using a standardized protocol provides an opportunity for dissociating the therapeutic effects of potential therapeutic agents and/or the biology of different aspects of inflammatory colitis.

## Supporting information

S1 Fig Differential DSS-induced gross and histopathology by various commonly used 4% DSS protocols. A) Experimental schematic representation for 3 additional DSS protocol time variations used: 4 days DSS exposure with or without reversal to regular drinking water for 3 days (R) and 7 days DSS exposure with reversal to water for 3 days. B) Percent body weight change from baseline for animals exposed to 4% DSS for the indicated time periods with either reversal to regular drinking water (R) for 3 days or not. Colon C) length and D) weight from animals exposed to regular drinking water or 4% DSS for various durations. E) H&E stained distal colon sections demonstrating mucosa architectural damage and immune cell infiltration after 4 and 7 days of DSS exposure followed by 3 day reversal to water. F) Distal colon wall thickness and G) mucosa layer/crypt length after exposure to the various DSS protocols. Data are reported as group means ± SEM. Asterisks correspond to p-values as follow: * p<0.05, **p<0.01 & ***p<0.001. Sample size for each group is indicated within its bar on the bar graph.
(TIF)

S2 Fig Rating scales used for the subjective assessment of distal colon inflammation and goblet cell loss. Description of typical pathology corresponding to each score on subjective rating scales for A) inflammation and B) goblet cell loss with representative images for each score.
(TIF)

S3 Fig Differential response in the open field by mice exposed to 4% DSS for 4 days or 7 days with or without reversal to water. A) Total number of rears after 60 minutes in the open field test revealed significant suppression in mice exposed to DSS for 4 and 7 days and then reverted to water for 3 extra days, but not in mice exposed to DSS for 4 days. B) Assessment of total ambulation revealed that, of the groups tested, only the 4 day DSS plus 3 day reversal to water group exhibited decreased movement. C) Normalizing rears by dividing rear counts by overall movement revealed that only the 7 day DSS plus 3 day reversal group exhibited decreased normalized rears. D) Computation of the average time spent per rear revealed that both 4 and 7 days of DSS exposure plus reversal to water, but not 4 day DSS exposure, caused significantly shorter rearing bouts than controls. Data are reported as group means ± SEM. Asterisks correspond to p-values as follow: *p<0.05, **p<0.01, ***p<0.001. Sample size for each group is indicated within its bar on the bar graph.
(TIF)

S4 Fig Colon inflammation was present by the 4^th^ day of DSS exposure and remained present even in mice that were reverted to regular drinking water for 3 days. A) Distal colon tissue expression levels of TNFα were significantly elevated after 4 days of exposure to DSS. B) IL-1β colon expression was greater in animals exposed to DSS for longer periods (7 days), and remained significantly elevated even after 3 days reversal to water. C) MPO and D) NPY expression levels were generally higher in mice exposed to DSS than controls and tended to be higher with longer exposure time. Data are reported as group means ± SEM. Asterisks correspond to p-values as follow: *p<0.05, **p<0.01, ***p<0.001. Sample size for each group is indicated within its bar on the bar graph.
(TIF)

S5 Fig Combined systemic treatment with the Y1 (BIBP 3226) and the Y2 (BIIE 0246) receptor inhibitors did not further improve DSS-induced pathology relative to either antagonist alone. All mice were exposed to a 4% DSS solution for 7 days and treated with either BIBP 3226, BIIE 0246 or a cocktail containing both antagonists injected once daily. A) Percent body weight change in DSS exposed mice demonstrated that Y2 receptor inhibition significantly slowed down weight loss progression. B) H&E stained distal colon sections demonstrating DSS-induced histopathology and immune cell infiltration. C) Colon length and D) muscle wall thickness were not altered by combined treatment with the antagonists. E) Rears normalized to locomotion were comparable between groups. F) Average rear duration was significantly decreased by Y1 receptor inhibition, increased by Y2 receptor inhibition and unchanged in the combined inhibitor treatment group. G) Distal colon tissue expression levels of TNFα were comparable between groups whereas H) IL-1β levels were significantly suppressed by Y1 receptor antagonism and to a comparable degree, in the group receiving the combined treatment. Similarly, L) DSS-stimulated colon MPO levels were significantly suppressed by independent and combined treatment of the Y1 and Y2 receptor inhibitors. J) Colon NPY expression was increased in the Y1 receptor inhibitor group but not the Y1 or the combined treatment groups. Data are reported as group means ± SEM. Asterisks correspond to p-values as follow: *p<0.05, **p<0.01, ***p<0.001. Sample size for each group is indicated within its bar on the bar graph.
(TIF)

S6 Fig Data for behavioral and inflammatory parameters unaltered by both NPY Y1 nor Y2 receptor inhibitors in DSS-exposed mice. In the open field task, neither total rears (A & E) nor total ambulations (B & F) were affected by either inhibitor treatment during DSS administration. In addition, neither MPO protein (C & G) nor TNF-α (D & H) were affected by either inhibitor. Data are reported as group means ± SEM. Significant p-values are illustrated as follow: * p<0.05, **p<0.01, ***p<0.001. Sample size for each group is indicated within its bar on the bar graph.
(TIF)

S7 Fig Statistical analysis results for each data figure. List of data figures and the corresponding statistical analyses completed for group comparisons.
(TIF)
