# Supplementary figures and images for: Pharmacological inhibition of NPY receptors illustrates dissociable features of experimental colitis in the mouse DSS model: Implications for preclinical evaluation of efficacy in an inflammatory bowel disease model
Source: PLoS One. 2019 Aug 1;14(8):e0220156. doi: 10.1371/journal.pone.0220156 (PMC6675069; doi:10.1371/journal.pone.0220156)

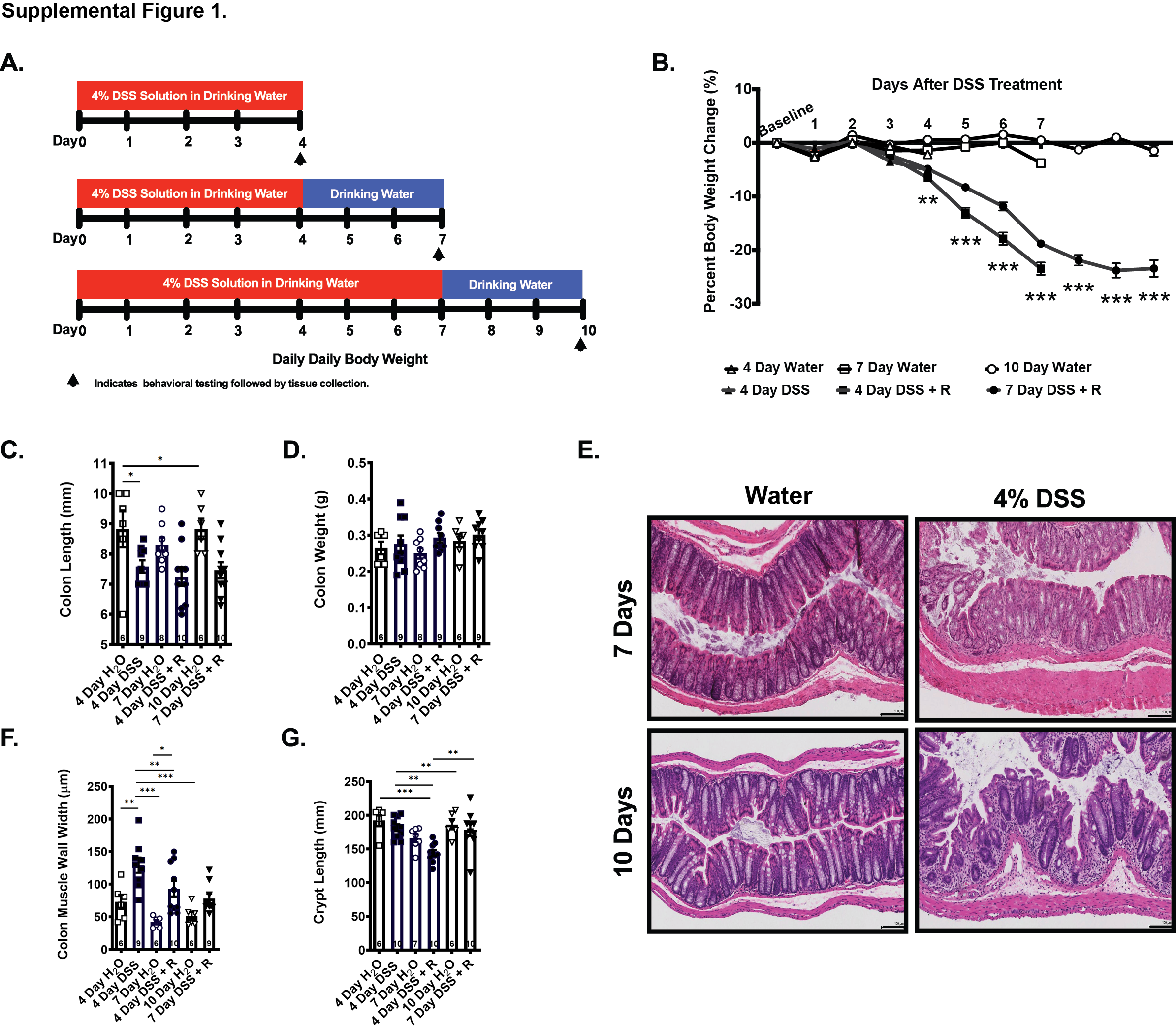

Supplement: S1 Fig — A) Experimental schematic representation for 3 additional DSS protocol time variations used: 4 days DSS exposure with or without reversal to regular drinking water for 3 days (R) and 7 days DSS exposure with reversal to water for 3 days. B) Percent body weight change from baseline for animals exposed to 4% DSS for the indicated time periods with either reversal to regular drinking water (R) for 3 days or not. Colon C) length and D) weight from animals exposed to regular drinking water or 4% DSS for various durations. E) H&E stained distal colon sections demonstrating mucosa architectural damage and immune cell infiltration after 4 and 7 days of DSS exposure followed by 3 day reversal to water. F) Distal colon wall thickness and G) mucosa layer/crypt length after exposure to the various DSS protocols. Data are reported as group means ± SEM. Asterisks correspond to p-values as follow: * p<0.05, **p<0.01 & ***p<0.001. Sample size for each group is indicated within its bar on the bar graph. (TIF) [file pone.0220156.s001.tif]

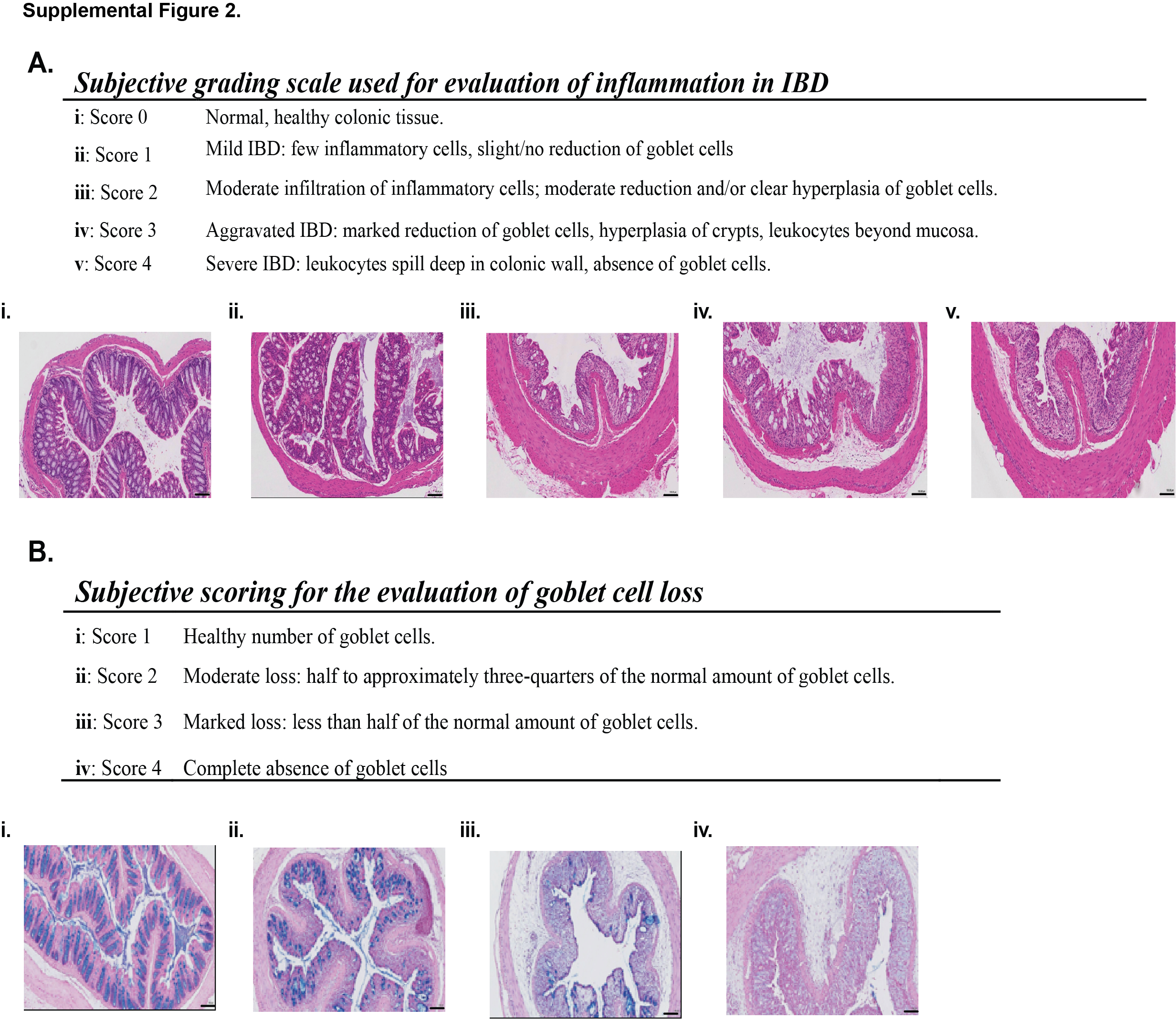

Supplement: S2 Fig — Description of typical pathology corresponding to each score on subjective rating scales for A) inflammation and B) goblet cell loss with representative images for each score. (TIF) [file pone.0220156.s002.tif]

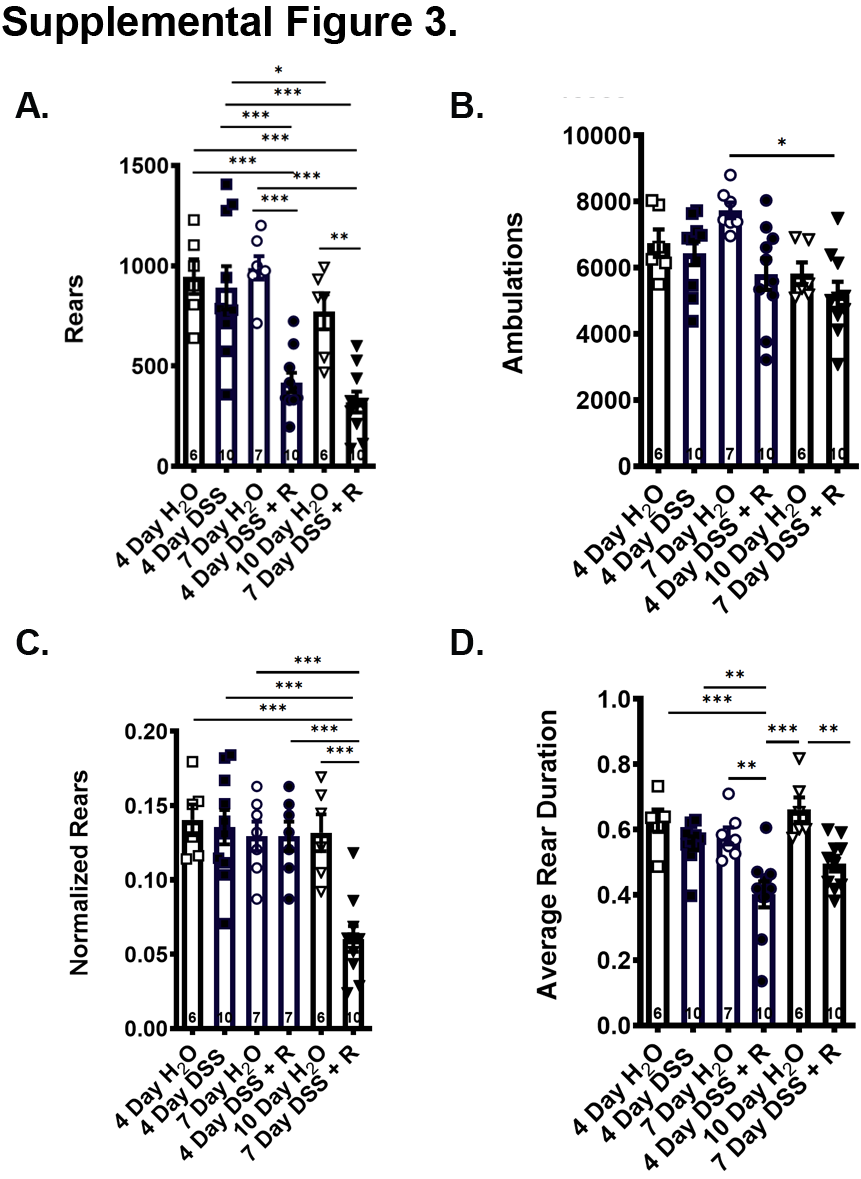

Supplement: S3 Fig — A) Total number of rears after 60 minutes in the open field test revealed significant suppression in mice exposed to DSS for 4 and 7 days and then reverted to water for 3 extra days, but not in mice exposed to DSS for 4 days. B) Assessment of total ambulation revealed that, of the groups tested, only the 4 day DSS plus 3 day reversal to water group exhibited decreased movement. C) Normalizing rears by dividing rear counts by overall movement revealed that only the 7 day DSS plus 3 day reversal group exhibited decreased normalized rears. D) Computation of the average time spent per rear revealed that both 4 and 7 days of DSS exposure plus reversal to water, but not 4 day DSS exposure, caused significantly shorter rearing bouts than controls. Data are reported as group means ± SEM. Asterisks correspond to p-values as follow: *p<0.05, **p<0.01, ***p<0.001. Sample size for each group is indicated within its bar on the bar graph. (TIF) [file pone.0220156.s003.tif]

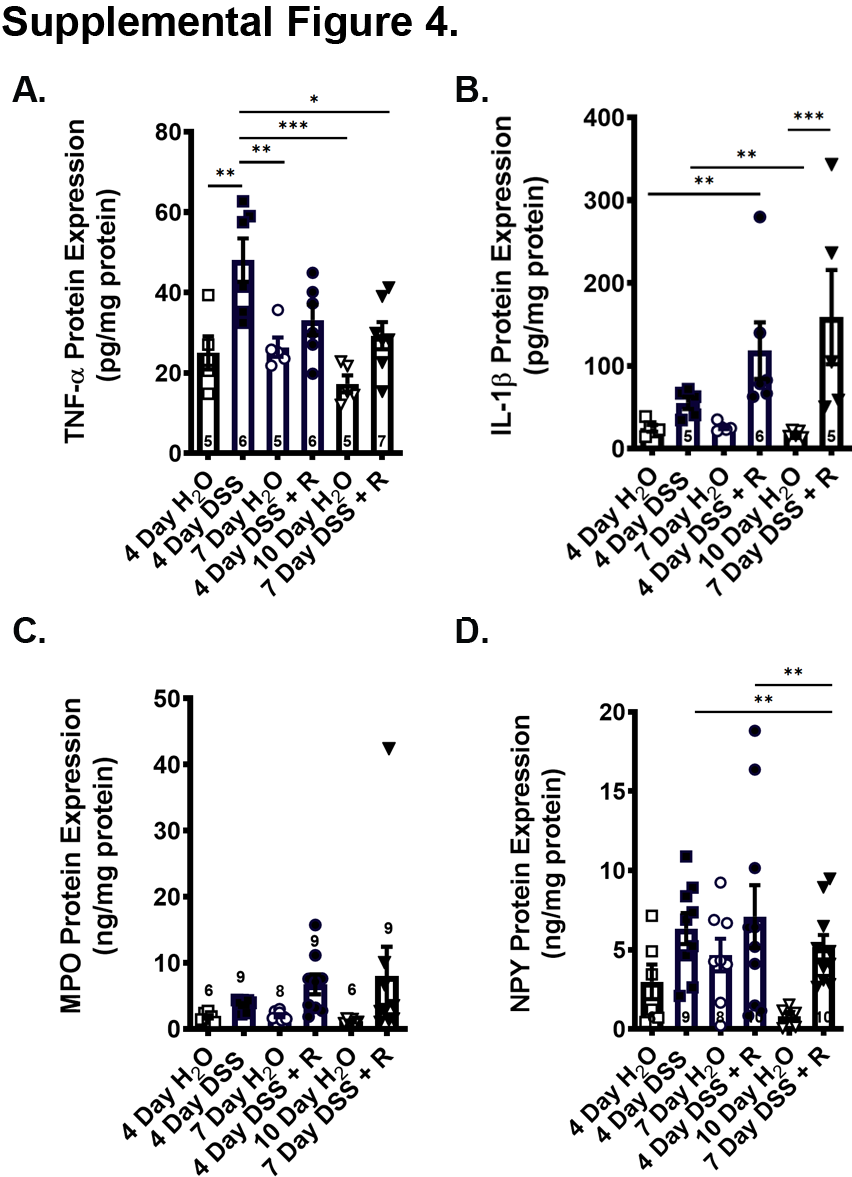

Supplement: S4 Fig — A) Distal colon tissue expression levels of TNFα were significantly elevated after 4 days of exposure to DSS. B) IL-1β colon expression was greater in animals exposed to DSS for longer periods (7 days), and remained significantly elevated even after 3 days reversal to water. C) MPO and D) NPY expression levels were generally higher in mice exposed to DSS than controls and tended to be higher with longer exposure time. Data are reported as group means ± SEM. Asterisks correspond to p-values as follow: *p<0.05, **p<0.01, ***p<0.001. Sample size for each group is indicated within its bar on the bar graph. (TIF) [file pone.0220156.s004.tif]

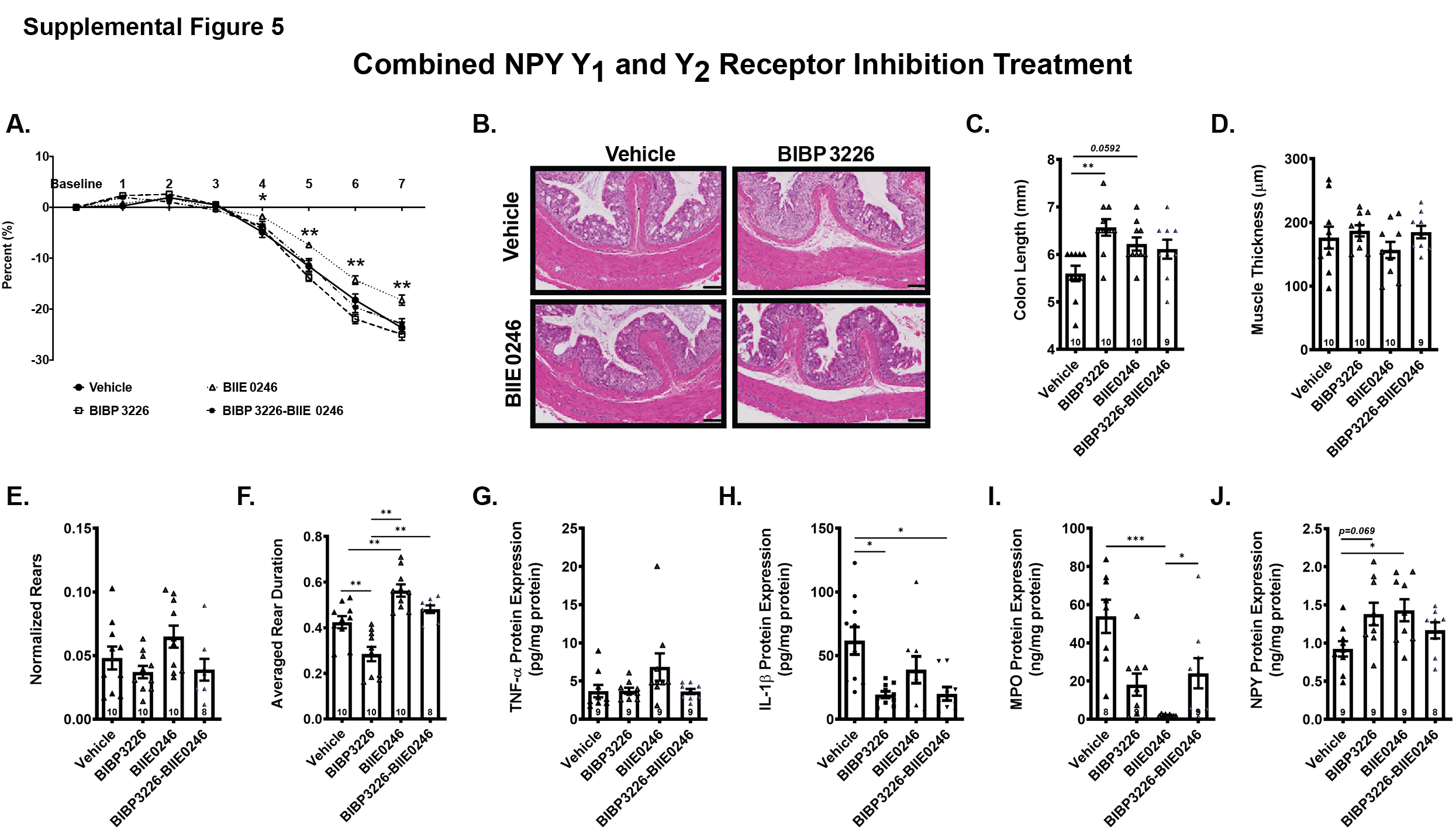

Supplement: S5 Fig — All mice were exposed to a 4% DSS solution for 7 days and treated with either BIBP 3226, BIIE 0246 or a cocktail containing both antagonists injected once daily. A) Percent body weight change in DSS exposed mice demonstrated that Y2 receptor inhibition significantly slowed down weight loss progression. B) H&E stained distal colon sections demonstrating DSS-induced histopathology and immune cell infiltration. C) Colon length and D) muscle wall thickness were not altered by combined treatment with the antagonists. E) Rears normalized to locomotion were comparable between groups. F) Average rear duration was significantly decreased by Y1 receptor inhibition, increased by Y2 receptor inhibition and unchanged in the combined inhibitor treatment group. G) Distal colon tissue expression levels of TNFα were comparable between groups whereas H) IL-1β levels were significantly suppressed by Y1 receptor antagonism and to a comparable degree, in the group receiving the combined treatment. Similarly, L) DSS-stimulated colon MPO levels were significantly suppressed by independent and combined treatment of the Y1 and Y2 receptor inhibitors. J) Colon NPY expression was increased in the Y1 receptor inhibitor group but not the Y1 or the combined treatment groups. Data are reported as group means ± SEM. Asterisks correspond to p-values as follow: *p<0.05, **p<0.01, ***p<0.001. Sample size for each group is indicated within its bar on the bar graph. (TIF) [file pone.0220156.s005.tif]

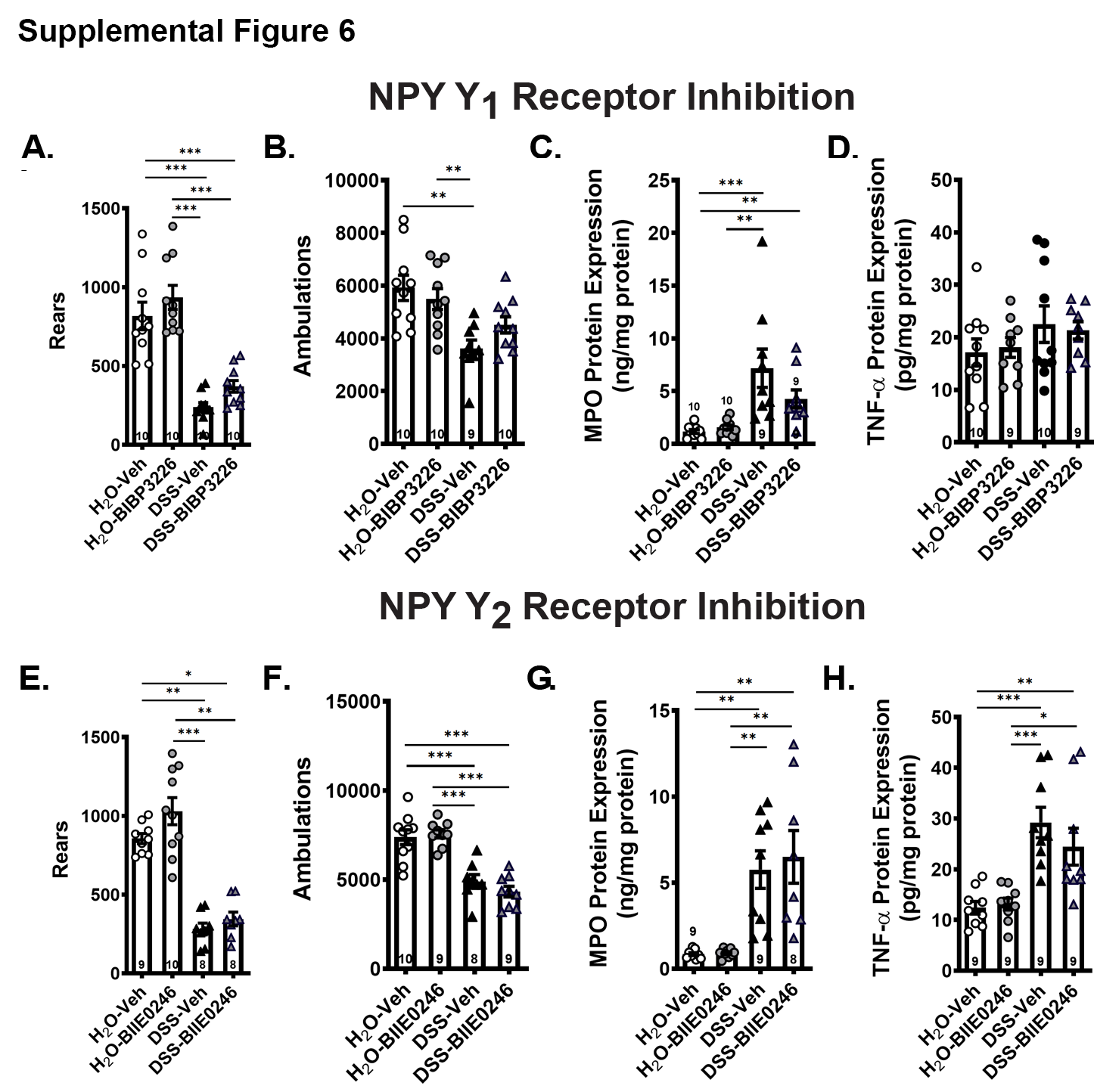

Supplement: S6 Fig — In the open field task, neither total rears (A & E) nor total ambulations (B & F) were affected by either inhibitor treatment during DSS administration. In addition, neither MPO protein (C & G) nor TNF-α (D & H) were affected by either inhibitor. Data are reported as group means ± SEM. Significant p-values are illustrated as follow: * p<0.05, **p<0.01, ***p<0.001. Sample size for each group is indicated within its bar on the bar graph. (TIF) [file pone.0220156.s006.tif]

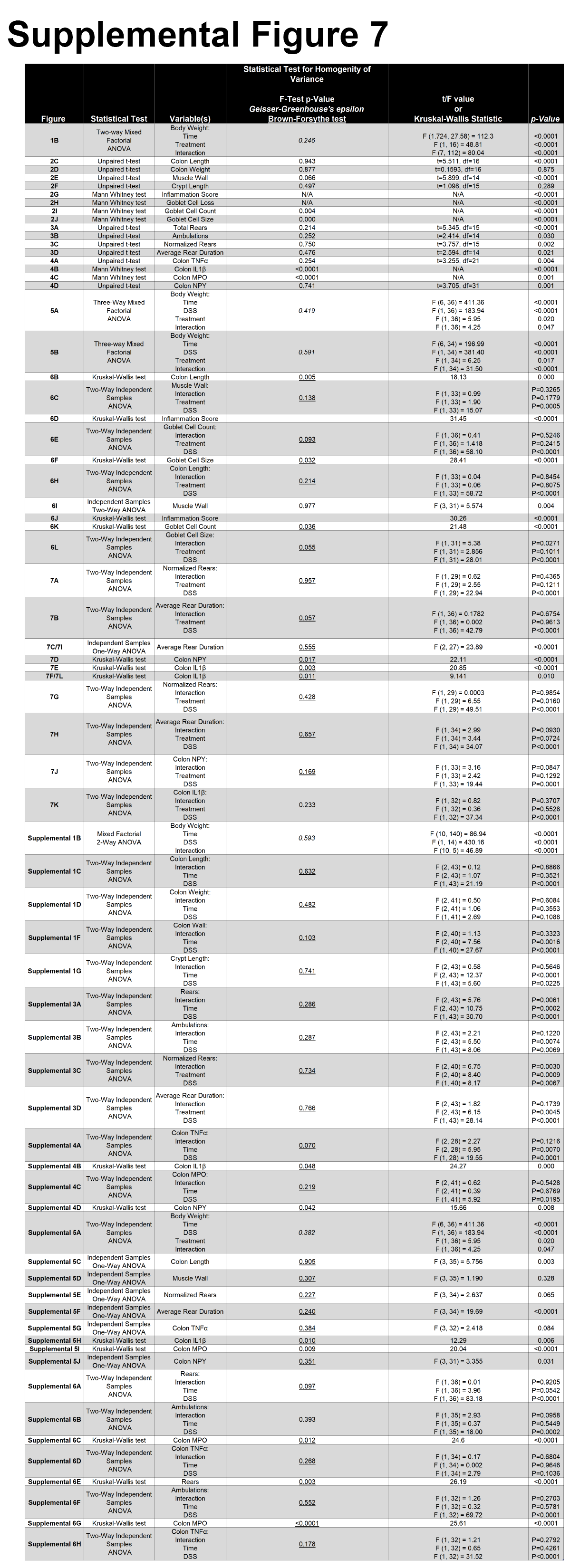

Supplement: S7 Fig — List of data figures and the corresponding statistical analyses completed for group comparisons. (TIF) [file pone.0220156.s007.tif]
